# Enantioselective pharmacokinetics of tramadol and its three main metabolites; impact of *CYP2D6*,* CYP2B6*, and *CYP3A4* genotype

**DOI:** 10.1002/prp2.419

**Published:** 2018-07-05

**Authors:** Pernilla Haage, Robert Kronstrand, Martin Josefsson, Simona Calistri, Ron H. N. van Schaik, Henrik Green, Fredrik C. Kugelberg

**Affiliations:** ^1^ Department of Forensic Genetics and Forensic Toxicology National Board of Forensic Medicine Linköping Sweden; ^2^ Department of Medical and Health Sciences Division of Drug Research Linköping University Linköping Sweden; ^3^ Department of Physics, Chemistry and Biology Linköping University Linköping Sweden; ^4^ Department of Clinical Chemistry Erasmus University Medical Center Rotterdam The Netherlands; ^5^ Scuola di Scienze della Salute Umana Università degli studi di Firenze Florence Italy

**Keywords:** *CYP2B6*, *CYP2D6*, *CYP3A4*, enantioselective pharmacokinetics, tramadol

## Abstract

Tramadol is a complex drug, being metabolized by polymorphic enzymes and administered as a racemate with the (+)‐ and (−)‐enantiomers of the parent compound and metabolites showing different pharmacological effects. The study aimed to simultaneously determine the enantiomer concentrations of tramadol, *O*‐desmethyltramadol, *N*‐desmethyltramadol, and *N,O*‐didesmethyltramadol following a single dose, and elucidate if enantioselective pharmacokinetics is associated with the time following drug intake and if interindividual differences may be genetically explained. Nineteen healthy volunteers were orally administered either 50 or 100 mg tramadol, whereupon blood samples were drawn at 17 occasions. Enantiomer concentrations in whole blood were measured by LC‐MS/MS and the *CYP2D6*,*CYP2B6* and *CYP3A4* genotype were determined, using the xTAG CYP2D6 Kit, pyrosequencing and real‐time PCR, respectively. A positive correlation between the (+)/(−)‐enantiomer ratio and time following drug administration was shown for all four enantiomer pairs. The largest increase in enantiomer ratio was observed for *N*‐desmethyltramadol in CYP2D6 extensive and intermediate metabolizers, rising from about two to almost seven during 24 hours following drug intake. CYP2D6 poor metabolizers showed metabolic profiles markedly different from the ones of intermediate and extensive metabolizers, with large area under the concentration curves (AUCs) of the *N*‐desmethyltramadol enantiomers and low corresponding values of the *O*‐desmethyltramadol and *N,O*‐didesmethyltramadol enantiomers, especially of the (+)‐enantiomers. Homozygosity of CYP2B6 *5 and *6 indicated a reduced enzyme function, although further studies are required to confirm it. In conclusion, the increase in enantiomer ratios over time might possibly be used to distinguish a recent tramadol intake from a past one. It also implies that, even though (+)‐*O*‐desmethyltramadol is regarded the enantiomer most potent in causing adverse effects, one should not investigate the (+)/(−)‐enantiomer ratio of *O*‐desmethyltramadol in relation to side effects without consideration for the time that has passed since drug intake.

**Abbreviations**
AUC: area under the concentration curve
DRS: drug‐related symptoms
EM: extensive metabolizer
IM: intermediate metabolizer
LOQ: limit of quantification
NDT: *N*‐desmethyltramadol
NODT: *N,O*‐didesmethyltramadol
ODT: *O‐desmethyltramadol*
PM: poor metabolizer
QCs: quality controls
RQCs: ratio quality controls
UM: ultrarapid metabolizer

## 1 INTRODUCTION

**Figure 1:**
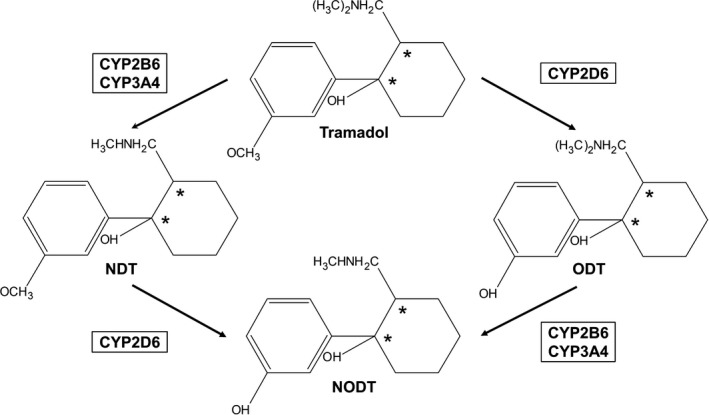
Tramadol metabolism. Three main metabolites are formed; *O*‐desmethyltramadol (ODT), *N*‐desmethyltramadol (NDT), and *N,O*‐didesmethyltramadol (NODT) by the CYP2D6, CYP2B6, and CYP3A4 enzymes. Asterisks indicate the two chiral centers

Tramadol is a relatively weak opioid with a dual mechanism of action, acting both as a μ‐opioid receptor agonist and a neurotransmitter reuptake inhibitor. Tramadol is administered as a racemate, with the (+)‐ and (−)‐enantiomers of the parent compound and their metabolites showing different pharmacological effects that synergistically accomplish pain relief.^1^ The (+)‐enantiomer of tramadol is most potent in inhibiting serotonin reuptake, while (−)‐tramadol preferentially inhibits noradrenaline reuptake.^2^ The (+)‐enantiomer of the metabolite *O*‐desmethyltramadol (ODT) is, however, exerting most of the opioid effects, having the highest affinity and highest potency to the μ‐opioid receptors.^3^ The general interpretation of previously performed and published studies on humans is that (+)‐ODT is the enantiomer most potent in relieving pain as well as in causing adverse effects.^4^ The tramadol enantiomers have, however, been proposed to cause adverse effects related to abuse and overdose of the drug.^5^ The CYP2D6 enzyme is accountable for the formation of ODT (Figure 1). Individuals may be classified as poor (PMs), intermediate (IMs), extensive (normal) (EMs) or ultrarapid metabolizers (UMs) according to the metabolic activity of the CYP2D6 enzyme, determined by either phenotyping or predicted from genotyping. Several studies have consistently showed that PMs, in comparison to EMs, have an increased exposure to (+)‐ and (−)‐tramadol combined with a reduced formation of especially (+)‐ODT but also of (−)‐ODT. UMs, on the contrary, are expected to form higher amounts of (+)‐ODT than EMs and to be more prone to opioid‐related adverse effects.^4^ Comparisons between EMs and UMs are, however, sparse in scientific literature. Apart from ODT, there are two additional primary tramadol metabolites, *N*‐desmethyltramadol (NDT), and *N,O*‐didesmethyltramadol (NODT) (Figure 1). NDT is pharmacologically inactive, while NODT may exert some opioid effects.^3^ Formation of NDT is mediated by the CYP2B6 and CYP3A4 enzyme, whereas the metabolism route of NODT is less assured. However, all three enzymes; CYP2D6, CYP2B6, and CYP3A4 are candidates (Figure 1).^6^ The *CYP2B6* gene, in similarity with *CYP2D6*, is highly polymorphic although less studied. The significance of *CYP2B6* polymorphisms in tramadol pharmacokinetics and pharmacodynamics has not yet been investigated. Nevertheless, both increased and decreased expression and activity of the enzyme as a result of genetic variation have been observed for other substrates.^7^ For CYP3A4, there is substantial interindividual variation in expression and function. Many polymorphisms have been identified in the *CYP3A4* gene, although most of them have not been associated with the phenotypical variability.^8^ However, studies indicate that CYP3A4*22 results in reduced enzyme activity, as demonstrated in vivo with the CYP3A4 probe drugs midazolam and erythromycin,^9^ and is affecting the pharmacokinetics and pharmacodynamics of several drugs.^10^, ^11^, ^12^

To better understand the pharmacological and adverse effects of tramadol, its metabolism and the influence of genetic polymorphisms, the use of enantioselective analytical methods and genotyping are of importance in studies of tramadol. Only two of the tramadol sterioisomers are administered as the racemic drug; the enantiomeric pair of 1R,2R (+) and 1S,2S (−).^13^ Tramadol and its three main metabolites thus give rise to four pairs of enantiomers in the blood following drug intake. To our knowledge, all eight compounds have not been simultaneously determined in a study population earlier. The overall aim of the present study was therefore to obtain such metabolic profiles in healthy volunteers receiving a single, therapeutic dose of tramadol. The enantioselective method needed for conducting such a study has previously been developed and validated.^14^ Specifically, the following research questions and hypotheses were posted:

1. Can interindividual differences in enantioselective metabolic profiles be explained by *CYP2D6, CYP2B6*, and/or *CYP3A4* genotype? The hypothesis is that interindividual differences are better explained by the combined genotype of all three enzymes involved in the metabolism of the drug, rather than by *CYP2D6* itself.
2. Is there a correlation between (+)/(−)‐enantiomer ratios of tramadol or its metabolites and time following drug administration? To our knowledge, this has not been investigated in a study population previously, although there are indicators of such a relation in literature. Rudaz et al. showed that enantiomer ratios of all four compounds in urine changed over time in one individual administered 100 mg tramadol.^15^

## 2 MATERIALS AND METHODS

### 2.1 Study population

The present study was based on analysis of blood samples collected in conjunction to a previous survey.^16^ Nineteen healthy volunteers aged between 19 and 34 years (median 25), were randomized into two groups, administered a single dose of either 50 or 100 mg tramadol as an oral immediate‐release formulation (Tramadol HEXAL, Sandoz). Blood samples were drawn from a peripheral venous catheter in the forearm at 17 occasions; prior to dosing and at 0.5, 1, 1.5, 2, 2.5, 3, 3.5, 4, 5, 6, 7, 8, 10, 24, 48, and 72 hours after administration. The tubes contained sodium fluoride and potassium oxalate. Two aliquots of each blood sample were stored at −80°C pending nonchiral^16^ and chiral analysis of tramadol and its metabolites, respectively.

The participants were not allowed to undergo any drug treatment, with the exception of contraceptive medication, which was used by seven subjects. However, one participant (subject 04), reported intake of an antihistamine about 12.5 hours before the tramadol administration. In conjunction to the last blood sampling on the first day (10 hours), the subjects were requested to fill in a form regarding their experience of drug‐related symptoms (DRS). The participants were asked to grade their experiences of nausea, dizziness, headache, vomiting, dry mouth, sweating, fatigue, and any other DRS on a scale between zero to five, where zero was no symptoms at all and five was the worst imaginable symptoms. The study was approved by the Regional Ethical Review Board in Linköping (No: 2011/337‐31), and written informed consent was gathered from the participants.

### 2.2 Pharmacokinetic assessments

The enantioselective analysis of tramadol, ODT, NDT, and NODT in whole blood was performed at the National Board of Forensic Medicine in Linköping, Sweden, using a validated LC‐MS/MS‐method that has been described in detail previously.^14^ In brief, blood samples of 0.5 g were fortified with an internal standard consisting of tramadol‐^13^C‐D_3_ and *O*‐desmethyl‐cis‐tramadol‐D_6_, followed by liquid–liquid extraction at pH 11. The LC‐MS/MS‐method operated in the reversed phase mode, using a chiral alpha‐1‐acid glycoprotein (AGP) column preceded by an AGP guard column. The mobile phase consisted of 0.8% acetonitrile and 99.2% ammonium acetate (20 mmol/L, pH 7.2). A postcolumn infusion with 0.05% formic acid in acetonitrile was used to enhance sensitivity. The calibration curve of each substance covered an enantiomer concentration range of 0.25‐250 ng/g. The limit of quantification (LOQ) was 0.125 ng/g for all enantiomers except for (+)‐ and (−)‐ODT with a corresponding limit of 0.50 ng/g. Historical calibration was utilized for the calculation of sample concentrations. All blood samples drawn from the same individual were analyzed in a separate run, together with at least four quality controls covering both enantiomeric quantitation (QCs) and enantiomeric ratio quantitation (RQCs). QCs were prepared from racemic reference compounds at enantiomer concentrations of 1 ng/g and 200 ng/g for all eight compounds. The QCs were not allowed to deviate from the nominal value with more than ±20% for approval of the run in question. The RQCs were prepared from pure enantiomeric reference compounds, in (+)/(−)‐enantiomer ratios of 0.25 and 4.0, respectively. The area ratio between the (+)‐ and (−)‐enantiomers in the RQCs was not allowed to deviate from the median area ratio obtained in the validation process with more than ±8% (two standard deviations). Long‐term stability tests at a low (1 ng/g) and a high (200 ng/g) enantiomer concentration were performed in −80°C up to 24 months. The difference in mean concentration between the stability samples and the fresh control samples at 24 months was in the range of −6% to −11% at the low concentration level and 0.5%‐5% at the high concentration level. The predetermined limit of acceptance was ±25%.

### 2.3 Genetic assessments

Genomic DNA was extracted using the Arrow Blood DNA 500 μL kit (DiaSorin, Saluggia, Italy) and a NorDiag Arrow instrument (Autogen, Holliston, MA). The DNA samples were stored at −20°C pending analysis. Genotyping was performed at the National Board of Forensic Medicine in Linköping, Sweden and at Erasmus University Medical Center in Rotterdam, The Netherlands.

For *CYP2D6*, gene multiplication and 15 alleles were investigated; *2 (−1584 C > G, 1661 G > C, 2850 C > T, 4180 G > C), *3 (2549delA), *4 (100 C > T, 1661 G > C, 1846 G > A, 2850 C > T, 4180 G > C), *5 (whole‐gene deletion), *6 (1707delT, 4180 G > C), *7 (2935 A > C), *8 (1661 G > C, 1758 G > T, 2850 C > T, 4180 G > C), *9 (2613delAGA), *10 (100 C > T, 1661 G > C, 4180 G > C), *11 (883 G > C, 1661 G > C, 2850 C > T, 4180 G > C), *15 (137_138insT), *17 (1023 C > T, 1661 G > C, 2850 C > T, 4180 G > C), *29 (1659 G > A, 1661 G > C, 2850 C > T, 3183 G > A, 4180 G > C), *35 (−1584 C > G, 31 G > A, 1661 G > C, 2850 C > T, 4180 G > C), *41 (1661 G > C, 2850 C > T, 2988 G > A, 4180 G > C), using the FDA approved xTAG CYP2D6 Kit v3 (Luminex, Austin, TX). Allele *3, *4, *5 and *6, as well as gene multiplication have been investigated also previously, using pyrosequencing technology.^16^ There were no discrepancies between the two different methodologies used. There is no true consensus on how to perform the classification of certain genotypes into phenotypes.^4^, ^17^, ^18^ In the present study, the guidelines of the Dutch Pharmacogenetics Working Group (DPWG) were used, which are also internationally recognized (http://www.pharmgkb.org), have been published, and which are used for dose recommendations.^19^ Consequently, individuals with two functional *CYP2D6* alleles or one functional and one decreased functional allele were classified as EMs, individuals with one nonfunctional allele and one functional or decreased functional allele as IMs and individuals with two nonfunctional alleles as PMs. UMs were defined as individuals with *CYP2D6* multiplications, resulting in at least three functional *CYP2D6* alleles.

Pyrosequencing technology was utilized for the *CYP2B6* genotyping. Three polymorphisms were identified in the *CYP2B6* gene; 516 G > T (rs3745274), 785 A > G (rs2279343) and 1459 C > T (rs3211371), making it possible to determine allele *4 (785 A > G), *5 (1459 C > T), *6 (516 G > T and 785 A > G), *7 (516 G > T, 785 A > G and 1459 C > T) and *9 (516 G > T). Amplification of the extracted DNA was performed in a total volume of 10 μL consisting of 5 μL HotStarTaq Plus Master Mix (Qiagen, Hilden, Germany), 3.6 μL RNase‐free water (Qiagen), 0.2 μL of the forward and reverse primer (Invitrogen, Lidingö, Sweden) in a concentration of 20 μmol/L and 1 μL of genomic DNA in a concentration of at least 30 ng/μL. The polymerase chain reaction (PCR) was initialized at 95°C for 5 minutes, followed by 40 cycles of denaturation at 95°C for 30 seconds, annealing at 50°C (516 G > T) or 58°C (785 A > G and 1459 C > T) for 30 seconds and extension at 72°C for 30 seconds. Final elongation was performed at 72°C for 10 minutes before the reaction was finalized at 4°C. Pyrosequencing was performed as previously described,^16^ using the primer sequences and dispensation orders given in Falk et al.^20^ For CYP3A4*22, Taqman analysis was used as described earlier.^21^ Alleles without any of the investigated polymorphisms were designated as functional *wild‐type* alleles (*1).

### 2.4 Pharmacokinetic and statistical calculations

Pharmacokinetic and genetic assessments were successfully performed in the blood samples of all participants. However, concerning five participants, blood samples could not be obtained at all 17 time points. From four of the individuals, there was one blood sample that could not be drawn, and from one individual there were three samples missing. This was due to different reasons, for example, a not properly working peripheral venous catheter.

*C*_max_ was defined as the maximum measured concentration and *t*_max_ the time when *C*_max_ was achieved. Other pharmacokinetic parameters were calculated in Microsoft Excel 2013, the area under the concentration curve (AUC) by using the linear trapezoidal method. Log‐transformed concentrations in the elimination phase were used to determine the elimination rate constant, and subsequently t_1/2_ and the extrapolated area between the time of the last measured concentration above LOQ and infinity. The extrapolated area of one or several compounds exceeded 20% of the total AUC in five individuals. From the subject showing the highest extrapolated areas, between 24% and 50% for all eight compounds, blood samples could not be drawn past 10 hours. The other four individuals presented extrapolated areas exceeding 20% regarding one or two compounds, the highest value being 27%.

Two statistical calculations, with a significance level of 0.05, were performed to elucidate the most appropriate approach for data presentation. Regarding the dose‐dependent parameters AUC and *C*_max_, a two‐tailed Wilcoxon rank‐sum test did not show any statistically significant difference between the 50 mg dosage group when obtained values were multiplied by two, and the 100 mg dosage group, indicating linear pharmacokinetics. Therefore, AUC values were dose‐adjusted to 100 mg prior to the establishment of Figure 3 and 4. A two‐tailed Wilcoxon rank‐sum test was also used to compare the enantiomer concentration ratios between the 50 and 100 mg dosage group at all time points. No statistically significant difference was detected. Consequently, the enantiomer ratios in Figure 5 are presented without distinction between the two dosage groups. Otherwise, no statistical tests were utilized, due to the relatively small sample size, and the results are instead descriptively presented. However, a two‐tailed Wilcoxon rank‐sum test with Bonferroni correction was used to compare the AUC values of tramadol in CYP2D6 EMs, IMs and PMs. The statistical tests were performed in MATLAB R2017b.

## 3 RESULTS

### 3.1 Metabolic profiles

**Figure 2:**
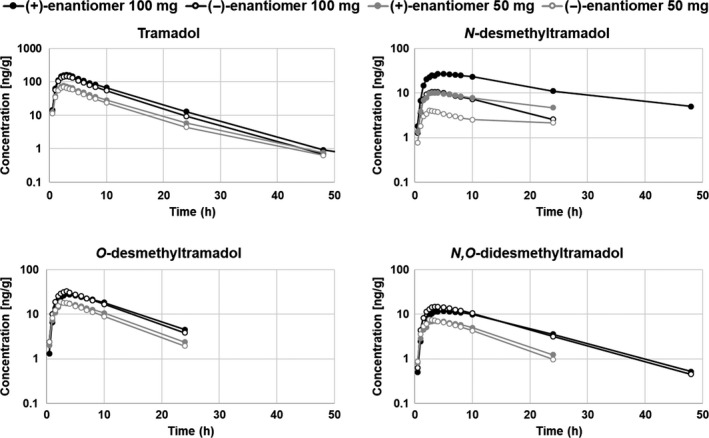
Mean enantiomer concentration versus time of tramadol and its three main metabolites following a single, oral dose of either 50 mg (n = 10) or 100 mg (n = 9). Only concentrations above the limit of quantification (LOQ) were included in the analysis, and the mean values presented are based on at least three individual observations

**Figure 3:**
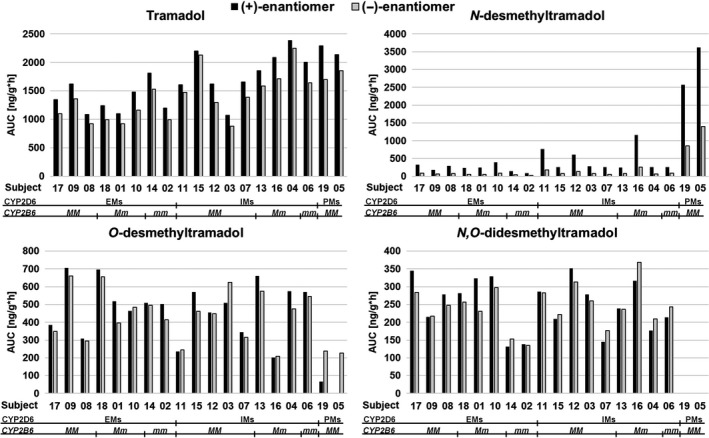
Metabolic profiles showing the total area under the concentration curve (AUC) of the enantiomers of tramadol and its three main metabolites in individuals with different CYP2D6 phenotypes and *CYP2B6* genotypes. Participants were orally administered either 50 or 100 mg tramadol, however, the present values were dose‐adjusted to 100 mg since linear pharmacokinetics was shown. EMs  =  extensive metabolizers, IMs  =  intermediate metabolizers, PMs  =  poor metabolizers, *M * =  major (*wild‐type*) allele, *m * =  minor (variant) allele

The mean concentration vs time curves for the enantiomers of tramadol and its three main metabolites are shown in Figure 2. The pharmacokinetic parameters AUC, *C*_max_, *t*_max_ and t_1/2_ are given in Table 1, where wide ranges implicated large interindividual differences. Those differences are further depicted in Figure 3, showing the dose‐adjusted, individual AUC of all four enantiomer pairs, termed metabolic profiles.

**Table 1:** Pharmacokinetic parameters of the enantiomers of tramadol and its three main metabolites *O*‐desmethyltramadol (ODT), *N*‐desmethyltramadol (NDT), and *N,O*‐didesmethyltramadol (NODT), following an oral, single dose of either 50 mg (n = 10) or 100 mg (n = 9)

	50 mg						100 mg					
	Mean		Median		Range		Mean		Median		Range	
	(+)	(−)	(+)	(−)	(+)	(−)	(+)	(−)	(+)	(−)	(+)	(−)
Tramadol												
AUC (ng/g*h)	786	668	775	631	537‐1192	440‐1124	1784	1501	1810	1526	1242‐2288	993‐2129
*C*_max_ (ng/g)	82	75	82	73	64‐110	57‐96	176	160	176	159	134‐208	117‐190
*t*_max_ (h)	2.5	2.3	2.6	2.4	1.6‐3.1	1.6‐2.6	2.7	2.6	3.0	3.0	1.5‐3.7	1.5‐3.7
*t*_1/2_ (h)	5.8	5.4	5.3	4.9	4.5‐8.0	4.2‐7.4	6.0	5.4	5.9	5.5	4.6‐7.4	3.9‐7.1
ODT												
AUC (ng/g*h)	225^a^	222	253^a^	222	154‐353^a^	113‐330	420	408	456	447	65‐697	208‐656
*C*_max_ (ng/g)	19	19	19	23	13‐23	5‐24	28	33	34	31	4‐51	14‐59
*t*_max_ (h)	3.3	2.9	3.1	2.6	2.6‐4.1	2.1‐4.1	3.5	2.9	3.5	3.2	2.1‐5.1	1.6‐4.1
*t*_1/2_ (h)	6.8^a^	6.5	6.8^a^	6.0	5.8‐8.3^a^	4.3‐10.3	7.9	6.9	7.3	7.2	5.9‐10.6	5.3‐9.4
NDT												
AUC (ng/g*h)	293	99	128	36	46‐1812	13‐698	700	194	324	84	145‐2569	44‐857
*C*_max_ (ng/g)	12	5	10	4	4‐33	2‐14	30	12	31	10	11‐60	5‐30
*t*_max_ (h)	5.4	3.5	3.6	2.6	2.6‐23.9	2.1‐10.1	5.5	3.8	4.1	3.6	1.5‐10.3	1.5‐8.1
*t*_1/2_ (h)	8.1	6.2	6.5	4.9	5.8‐21.7	2.9‐17.8	8.2	6.1	7.4	5.7	4.8‐18.5	3.8‐10.8
NODT												
AUC (ng/g*h)	105^a^	112^a^	107^a^	115^a^	69‐164^a^	68‐149^a^	270^a^	264^a^	283^a^	270^a^	131‐352^a^	152‐369^a^
*C*_max_ (ng/g)	7	8	7	8	4‐11	0.7‐13	13	16	13	16	0.3‐25	5‐27
*t*_max_ (h)	4.2	5.3	3.6	2.9	2.6‐8.1	2.6‐23.9	6.1	5.0	5.3	4.1	3.5‐10.3	1.5‐10.3
*t*_1/2_ (h)	7.7^a^	6.7^a^	7.5^a^	6.3^a^	6.6‐9.2^a^	5.3‐8.5^a^	7.7^a^	6.7^a^	7.9^a^	6.6^a^	5.8‐9.8^a^	5.0‐8.8^a^

a Indicate that the results of the CYP2D6 poor metabolizers were excluded, since values were below LOQ or could not be calculated.

The metabolic profiles of the two CYP2D6 PMs, subject 05 and 19, were markedly different compared to the ones of individuals with other CYP2D6 phenotypes (Figure 3). They were characterized by large AUCs of the NDT enantiomers, with concomitant small AUCs of the ODT and NODT enantiomers. The (+)‐enantiomers of ODT and NODT were affected to a larger extent than the (−)‐enantiomers. Subject 05, administered the 50 mg dose, never achieved (+)‐ODT or (+)‐NODT concentration levels above LOQ. Concentrations above LOQ of the NODT enantiomers in the two PMs were low and constituted more of a plateau over time than a curve. Total AUC and t_1/2_ could therefore not be calculated. On group‐level, both PMs and IMs showed larger AUCs of the tramadol enantiomers compared to the EMs (Figure 3), with *P*‐values ranging from 0.021 to 0.044. However, the differences were not statistically significant after correction for multiple comparisons, since significance then required a *P*‐value below 0.017.

In the subjects not being CYP2D6 PMs, ODT was the major metabolite with AUCs larger than that of the NDT and NODT enantiomers (Figure 3). Three CYP2D6 IMs, however, constituted an exception, most prominent in subject 16. This individual showed larger AUCs of both the NDT and NODT enantiomers than of the ODT enantiomers. Subject 16 did also report the highest DRS score (which has been fully declared previously^16^), and did both faint (at 1 hour and 15 minutes following drug intake) and vomit (at about 4 hours following drug administration and also later in the evening) during the experimental day. Subject 16 carried one decreased functional *CYP2D6* allele and one nonfunctional *CYP2D6* allele (Table 2).

**Table 2:** *CYP2D6*,* CYP2B6*, and *CYP3A4* genotype of 19 healthy volunteers administered either 50 or 100 mg tramadol

CYP2D6 phenotype	Subject	Dose	*CYP2D6*	*CYP2B6*	*CYP3A4*
EM	17	100	*1/*9	*1/*1	*1/*1
EM	09	50	*1/*1	*1/*1	*1/*1
EM	08	50	*1/*41	*1/*1	*1/*1
EM	18	100	*2/*35	*1/*5	*1/*22
EM	01	50	*1/*2	*1/*5	*1/*1
EM	10	50	*2/*41	*1/*5	*1/*1
EM	14	100	*1/*35	*5/*5	*1/*1
EM	02	50	*1/*1	*6/*6	*1/*1
IM	11	100	*1/*5	*1/*1	*1/*1
IM	15	100	*1/*4	*1/*1	*1/*1
IM	12	100	*4/*35	*1/*1	*1/*1
IM	03	50	*1/*5	*1/*1	*1/*1
IM	07	50	*2/*5	*1/*1	*1/*1
IM	13	100	*1/*3	*1/*7	*1/*1
IM	16	100	*4/*41	*1/*6	*1/*1
IM	04	50	*1/*5	*1/*6	*1/*1
IM	06	50	*1/*4	*6/*6	*1/*1
PM	19	100	*4/*4	*1/*1	*1/*1
PM	05	50	*4/*5	*1/*1	*1/*1

Large AUCs of the ODT enantiomers in relation to the AUCs of the tramadol enantiomers were shown in subject 18 and 03 (Figure 3). The AUC (+)‐ODT/(+)‐tramadol ratio in those individuals was 0.56 and 0.47, respectively, with a mean value in the whole group of 0.30. The AUC (−)‐ODT/(−)‐tramadol ratio was 0.66 and 0.71, respectively, with a mean ratio of 0.33. Subject 18 was a CYP2D6 EM with *CYP2B6* genotype *1/*5 and *CYP3A4* genotype *1/*22. Subject 03 was a CYP2D6 IM with no other of the investigated polymorphisms detected (Table 2).

**Figure 4:**
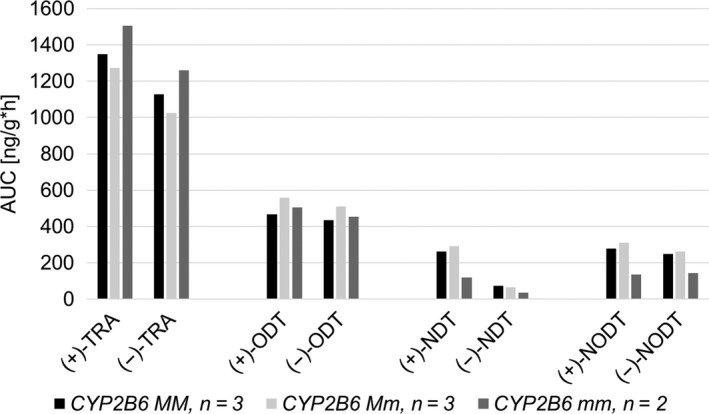
Mean area under the concentration curves (AUCs) of the enantiomers of tramadol and its three main metabolites in CYP2D6 extensive metabolizers, having different *CYP2B6* genotypes. AUC values were dose‐adjusted to 100 mg. *M * =  major (*wild‐type*) allele, *m * =  minor (variant) allele, TRA = Tramadol, ODT = *O*‐desmethyltramadol, NDT = *N*‐desmethyltramadol, NODT = *N,O*‐didesmethyltramadol

Two other individuals, subject 02 and 14, showed metabolic profiles consisting of the smallest AUCs of the NDT enantiomers and, with the exception of the CYP2D6 PMs, the smallest AUCs of the NODT enantiomers (Figure 3). Both individuals were CYP2D6 EMs with the *CYP2B6* genotype *6/*6 and *5/*5, respectively (Table 2). The differences in metabolic profiles of those two subjects compared to the other CYP2D6 EMs are further illustrated in Figure 4. Regarding both NDT and NODT, the mean AUC of the variant homozygotes was reduced by approximately 50% compared to the *wild‐type* homozygotes and the heterozygotes. Subject 06, being a CYP2D6 IM with the CYP2B6 *6/*6 genotype did, however, not show the same marked difference in metabolic profile.

### 3.2 Correlation between enantiomer ratios and time following drug administration

**Figure 5:**
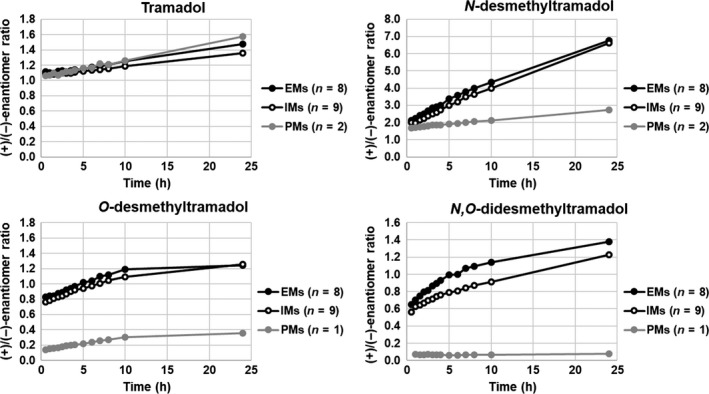
Correlation between the mean (+)/(−)‐enantiomer ratios of tramadol, *O*‐desmethyltramadol, *N*‐desmethyltramadol and *N,O*‐didesmethyltramadol, and time in CYP2D6 poor (PMs), intermediate (IMs) and extensive metabolizers (EMs), respectively. The healthy volunteers were administered an oral, single dose of either 50 or 100 mg tramadol. One of the two PMs never achieved concentrations of (+)‐*O*‐desmethyltramadol or (+)‐*N,O*‐didesmethyltramadol above the limit of quantification, and subsequently enantiomer ratios could not be calculated

Positive correlations were found when the mean (+)/(−)‐enantiomer ratios of tramadol and its main metabolites were plotted against time after drug intake in CYP2D6 PMs, IMs, and EMs (Figure 5). The correlations of tramadol and NDT were close to linear during 24 hours, while there was a change in linearity for ODT and NODT. The enantiomer ratios of tramadol were similar in all genotype groups, while the CYP2D6 PMs showed markedly lower ratios and also a smaller increase in enantiomer ratio over time regarding the metabolites. Concerning NODT, the PM did not show any change at all in enantiomer ratio. One of the CYP2D6 IMs differed from the others by having notably lower (+)/(−)‐enantiomer ratios of ODT and NODT, ratios that placed themselves between the IM and PM group (Data S1). This individual, subject 16, carried a nonfunctional allele in combination with a decreased functional one, while the other IMs carried one nonfunctional and one functional allele. The largest increase in enantiomer ratio over time was seen for NDT, with about three times increase in enantiomer ratio over 24 hours for the CYP2D6 EMs and IMs. However, in conformity with the results of the other pharmacokinetic parameters, there were large interindividual differences (Data S1).

## 4 DISCUSSION

### 4.1 Metabolic profiles

This study describes the enantioselective pharmacokinetics of tramadol and its three main metabolites simultaneously. Previously, only less numerous enantiomer pairs have been studied concurrently. The observed mean and median values of AUC, *C*_max_, *t*_max_ , and *t*_1/2_ regarding tramadol, ODT, and NDT are in accordance with previous studies that also administered a single, oral dose of 50^22^ and 100 mg.^22^, ^23^, ^24^, ^25^ However, data available in literature reflects the pharmacokinetics in plasma and serum, while the present study presents the pharmacokinetics in whole blood. Enantiomeric pharmacokinetic parameters of NODT have previously only been reported following a 200 mg extended‐release formulation.^26^

Great interindividual differences in drug exposure were apparent, illustrated by the metabolic profiles of the four enantiomer pairs (Figure 3). Two CYP2D6 PMs participated in the present study, showing metabolic profiles different from the others. Conspicuously large AUCs of the NDT enantiomers and low corresponding values of the ODT and NODT enantiomers were shown. The (+)‐enantiomers of ODT and NODT were affected to a larger extent than the (−)‐enantiomers. Those results are in line with previous publications, showing that PMs, in comparison to EMs, achieve a decreased AUC of mainly (+)‐ODT but also of (−)‐ODT 22, 25, 27 and an increased AUC of the NDT enantiomers.^25^ However, enantioselective measurements of NODT have not, to our knowledge, been performed in different CYP2D6 phenotype groups previously. Considering the tramadol metabolism scheme (Figure 1), the observed reduced levels of the NODT enantiomers were expected. NODT is formed from both ODT and NDT. Since PMs only form low amounts of ODT due to the abolished function of CYP2D6, most NODT will be formed from NDT. It is proposed that the enzyme metabolizing NDT to NODT is also CYP2D6, for what reason only low concentrations of NODT, and especially of (+)‐NODT, are expected to be formed. As a consequence, the amounts of the NDT enantiomers will accumulate. Earlier studies have also shown that PMs achieve an increased AUC of both (+)‐ and (−)‐tramadol,^22^, ^25^, ^27^ and the present one indicated that both CYP2D6 IMs and PMs achieved larger AUCs of the tramadol enantiomers than the EMs.

Another metabolic profile that leapt out belonged to a CYP2D6 IM, the only one carrying a nonfunctional allele in combination with a decreased functional one. The AUCs of both the NDT and NODT enantiomers exceeded the ones of the ODT enantiomers. In accordance with the *CYP2D6* genotype, those results indicate a reduced, but not abolished, CYP2D6 function. The same individual was the one being mostly affected by the drug, both fainting and vomiting during the experimental day. The general hypothesis in literature regarding adverse effects following tramadol administration is that the frequency and intensity is related to the concentrations of (+)‐ODT. The higher the concentration, the higher risk of side effects and toxicity.^4^, ^6^ However, review authors have expressed a need of more studies before a relationship between CYP2D6 metabolizer status and tramadol adverse effects might be established.^28^, ^29^ In the present study, the individual experiencing most DRS was the one with the second lowest *C*_max_ and AUC of (+)‐ODT in the 100 mg dosage group (only the PM individual showed lower values). However, DRS should probably better be examined among clinical patients on tramadol treatment, than among healthy volunteers only receiving a single, therapeutic dose.

Metabolic profiles with large AUCs of the ODT enantiomers in relation to the AUCs of the tramadol enantiomers were shown in two subjects, and could be expected in CYP2D6 UMs. However, the current individuals were a CYP2D6 EM with *CYP2B6* genotype *1/*5 and *CYP3A4* genotype *1/*22, and a CYP2D6 IM, respectively. No other individual in the present study carried the CYP3A4*22 allele, an allele that has been associated with a decreased enzyme function.^9^ However, if a reduced *N*‐demethylation of tramadol, by indirect means, had caused the increased *O*‐demethylation, a smaller AUC of the NDT‐enantiomers might have been expected. Another possible explanation for increased AUC of the ODT enantiomers, although not investigated in the present study, is polymorphisms in the *SLC22A1* gene. The gene encodes a transporter named OCT1 that mediates reuptake of ODT in the liver. Polymorphisms resulting in an inactive protein have been shown to cause increased AUCs of (+)‐ODT and (−)‐ODT in healthy volunteers administered a single oral dose of 100 mg.^30^

The significance of *CYP2B6* polymorphisms in tramadol pharmacokinetics has not been carefully investigated. In a study that elucidated potential genetic factors and covariates affecting the clearance of (+)‐ and (−)‐tramadol in a group of neuropathic pain patients, CYP2B6 *9 was not found to significantly contribute.^31^ In the present study, the metabolic profiles with the smallest AUCs of the NDT enantiomers belonged to two individuals being CYP2D6 EMs with the *CYP2B6* genotype *6/*6 and *5/*5, respectively. They also showed small AUCs of the NODT enantiomers, only the PMs showed lower values. However, a gene–dose relationship, which is commonly shown for CYP enzymes, could not be demonstrated (Figure 4). Nevertheless, the difference, large or small, between *wild‐type* homozygotes and heterozygotes may be substrate dependent. Also, regarding this particular pharmacokinetic parameter, not only CYP2B6 but also CYP3A4, is expected to influence the outcome. Herein, the CYP2D6 IM with the CYP2B6 *6/*6 genotype did not show the same pronounced difference in metabolic profile, which further underlines the need of additional studies on this subject.

### 4.2 Correlation between enantiomer ratios and time following drug administration

A positive correlation between the (+)/(−)‐enantiomer ratio and time following drug administration was found regarding all four enantiomer pairs. If further investigated in a larger population, also taking different dosages, regular dosing and drug interactions into consideration, enantiomer ratios could potentially be used to estimate the time of tramadol intake, or with less nicety, distinguish between a recent or past administration. From a clinical and forensic perspective, that could, for instance, be helpful regarding patient adherence to medical treatment and in drugs and driving cases, respectively. However, from the present investigation, it is obvious that there are large interindividual differences, which must also be carefully considered in further studies. Nevertheless, an advantage with the proposed method is the possibility of combining the information of four different enantiomer ratios, given that the *CYP2D6* genotype is known.

Both the tramadol and ODT enantiomers have been subjects of discussion regarding adverse effects.^4^, ^5^, ^6^ It has been clarified that the tramadol enantiomers have different pharmacological effects, although it has not been examined if there is a difference also in their ability to produce side effects. For ODT, it is the (+)‐enantiomer that has been proposed to be most potent in causing adverse effects. In further investigations on the subject, it might be well worth knowing that the (+)/(−)‐enantiomer ratios increases with the time following ingestion.

## 5 CONCLUSIONS

The most significant finding of the study was that (+)/(−)‐enantiomer ratios of tramadol and its three main metabolites were positively correlated with the time following drug intake. If further investigated, the ratios might be used to distinguish a recent drug intake from a past one. Concerning the proposed association between concentrations of (+)‐ODT and the risk of adverse effects, it is important knowing that the (+)/(−)‐enantiomer ratios of ODT are affected not only by the *CYP2D6* genotype, but also by the time that has passed between drug administration and blood sampling. It was confirmed that CYP2D6 PMs show a metabolic profile significantly different from the ones of IMs and EMs. Considerably larger AUCs of the NDT enantiomers were found, combined with much smaller, or noncalculable, AUCs of the ODT and NODT enantiomers, especially of the (+)‐enantiomers. Homozygosity of the *CYP2B6* alleles *5 and *6, not previously investigated regarding tramadol metabolism, indicated a reduced enzyme function. However, this pilot finding needs to be confirmed in a larger study population.

## DISCLOSURE

The authors have nothing to disclose.
