# Supplementary material for: Enantioselective pharmacokinetics of tramadol and its three main metabolites; impact of CYP2D6, CYP2B6, and CYP3A4 genotype
Source: Pharmacol Res Perspect. 2018 Jul 5;6(4):e00419. doi: 10.1002/prp2.419 (PMC6034060; doi:10.1002/prp2.419)
Supplement: Supplementary file 1 [file PRP2-6-e00419-s001.pdf]

Supplementary Data

The diagrams show the correlation between the enantiomer ratios of tramadol, *O*-desmethyltramadol (ODT), *N*-desmethyltramadol (NDT) and *N,O*-didesmethyltramadol (NODT) and time following drug administration on an individual basis.

ENANTIOSELECTIVE PHARMACOKINETICS OF TRAMADOL AND ITS THREE MAIN METABOLITES; IMPACT OF *CYP2D6*, *CYP2B6* AND *CYP3A4* GENOTYPE

Haage et al.

Pharmacology Research & Perspectives

Subject 17

Subject 09

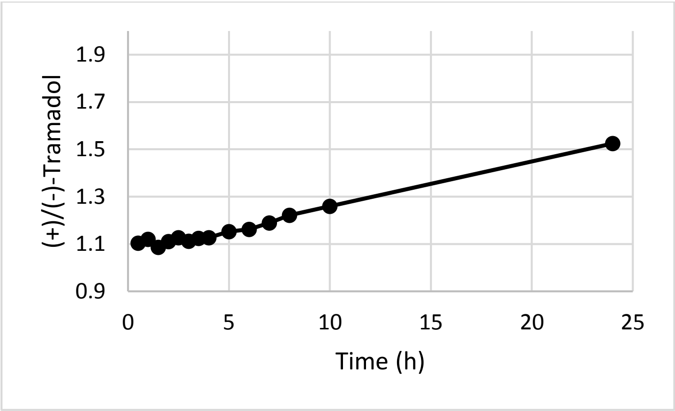

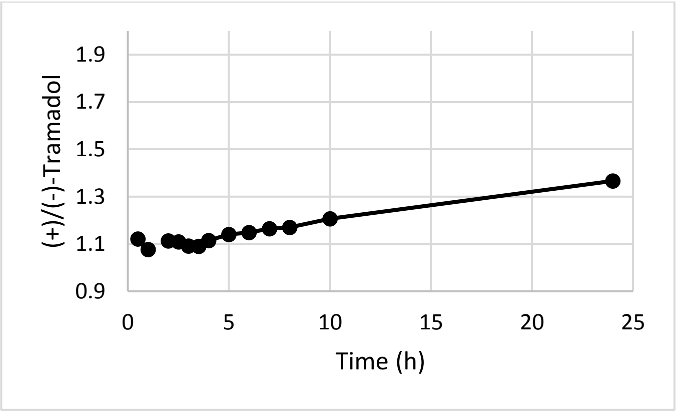

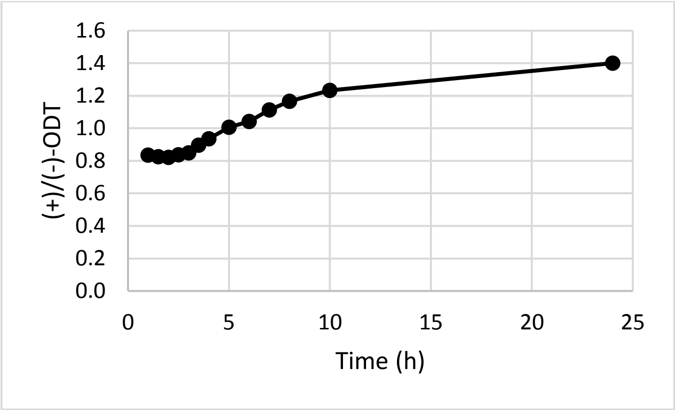

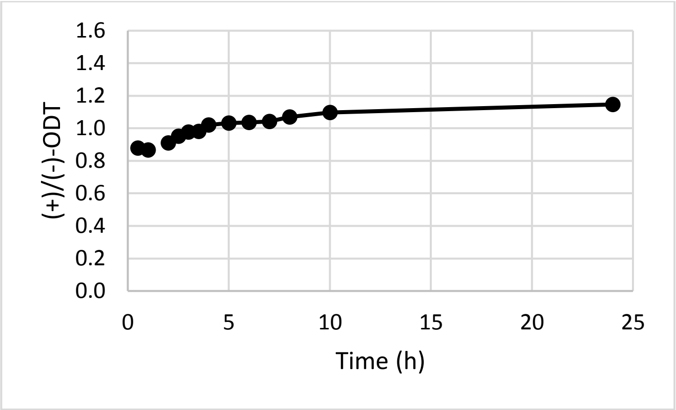

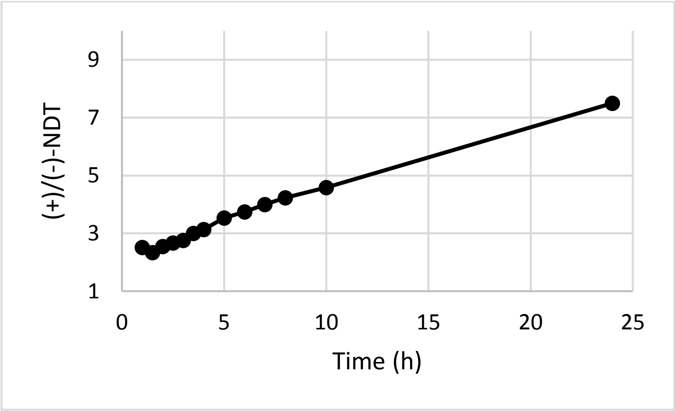

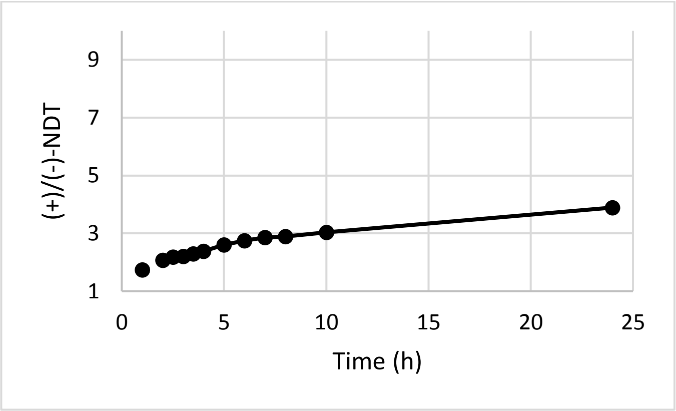

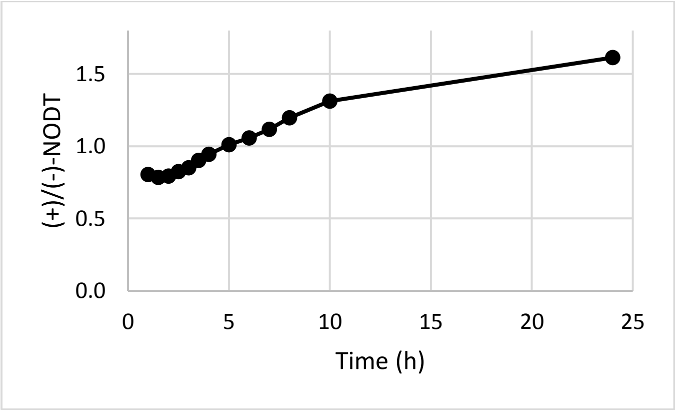

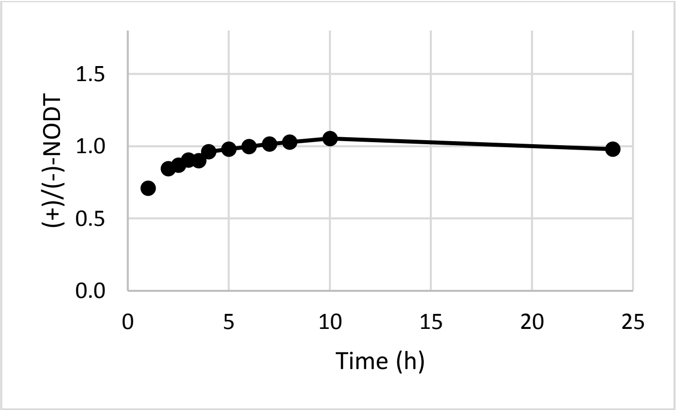

Subject 08

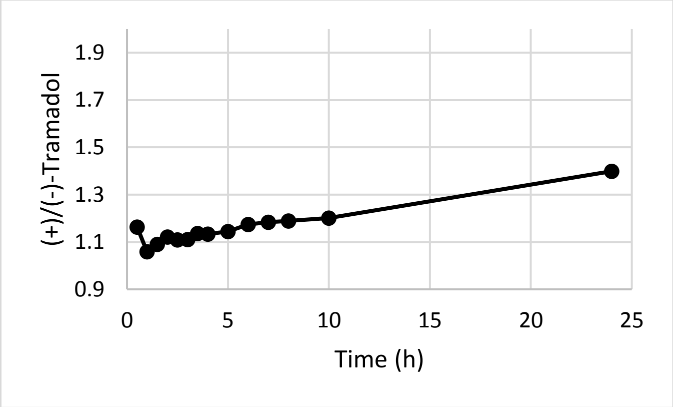

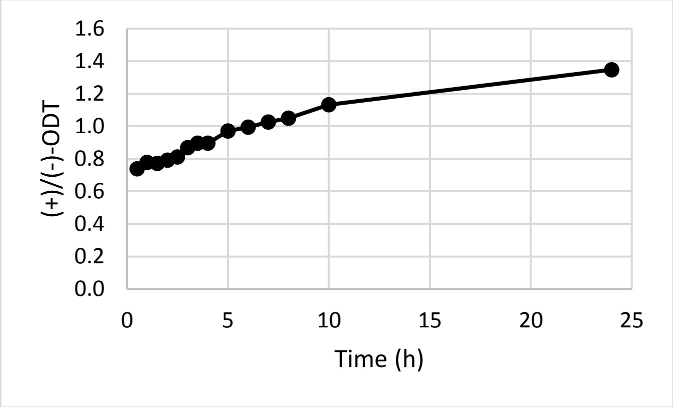

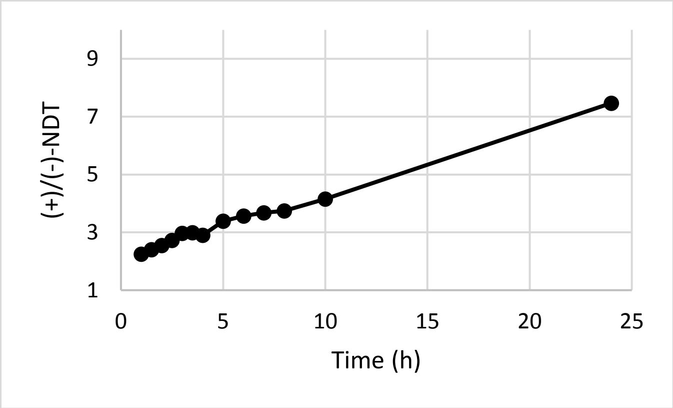

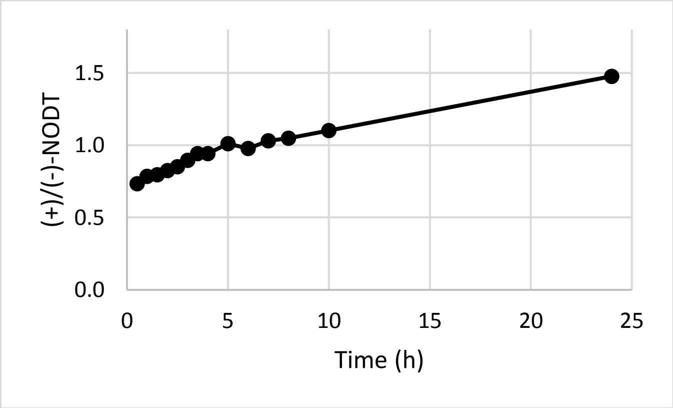

Subject 18

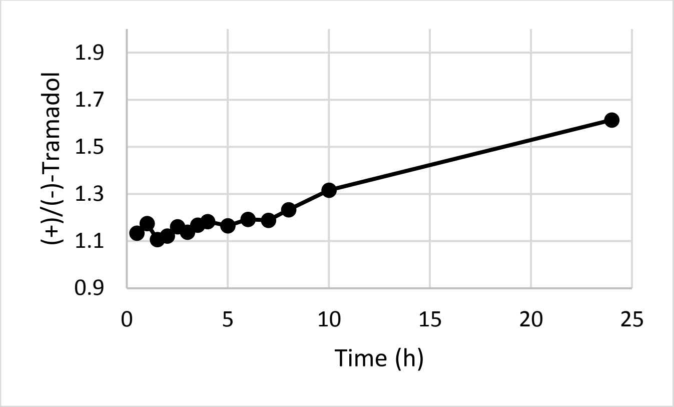

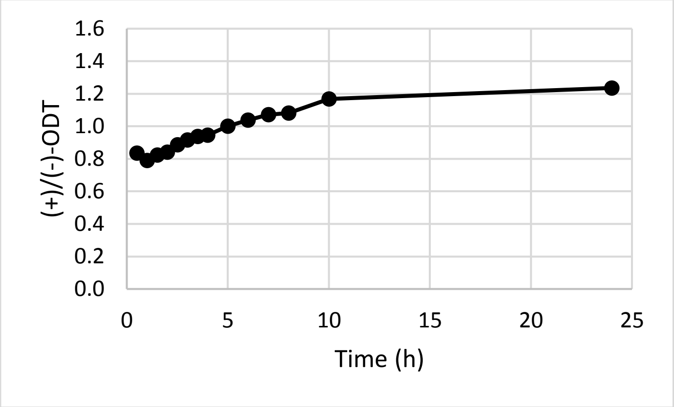

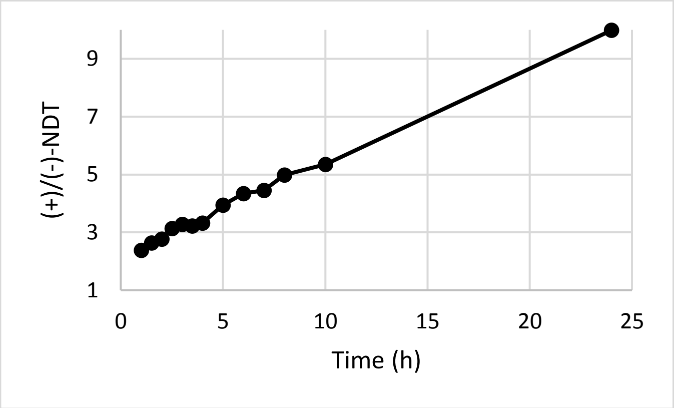

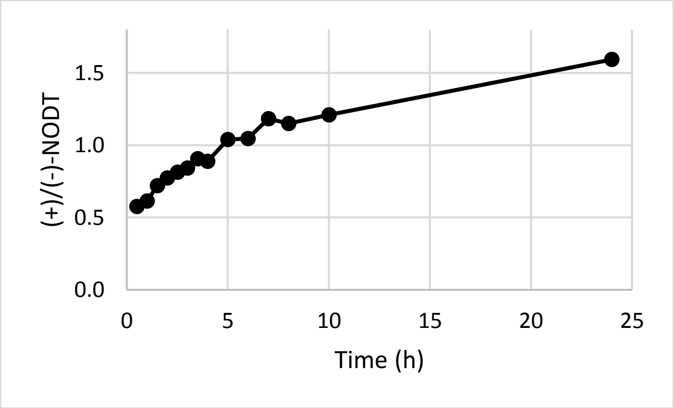

Subject 01

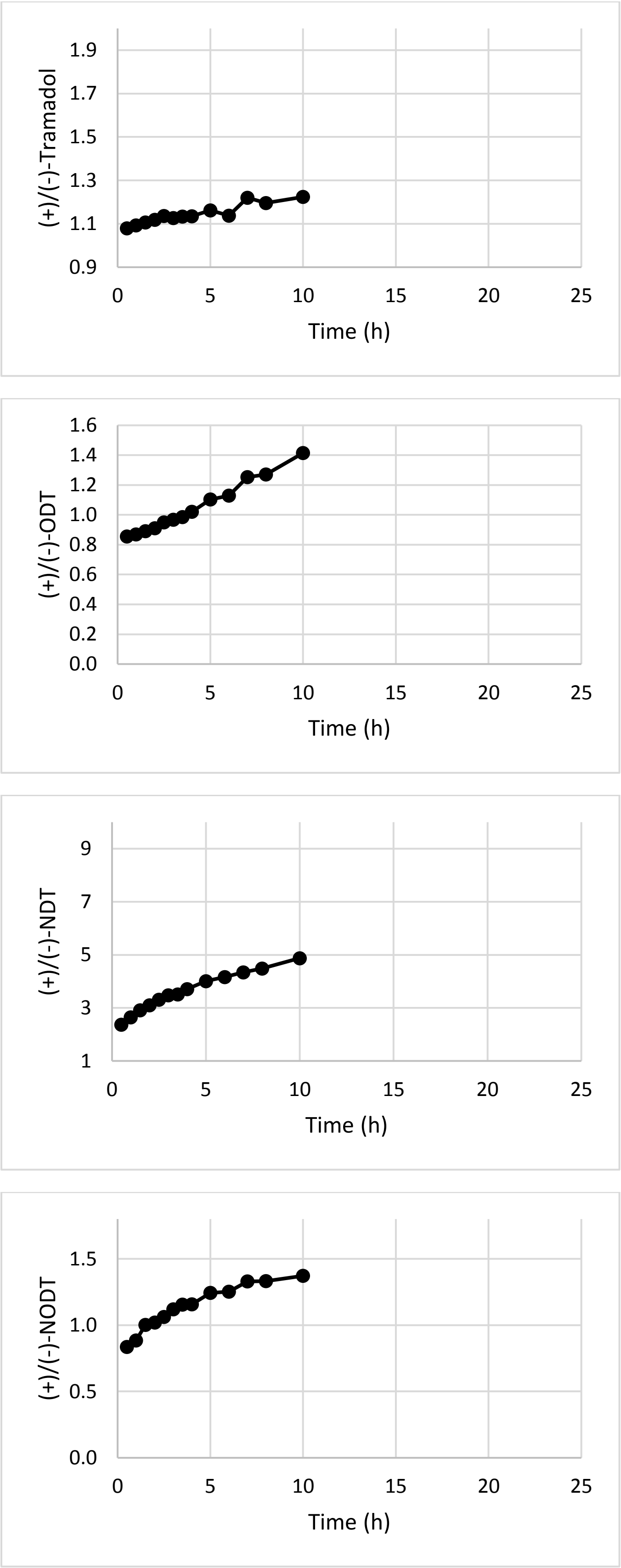

Subject 10

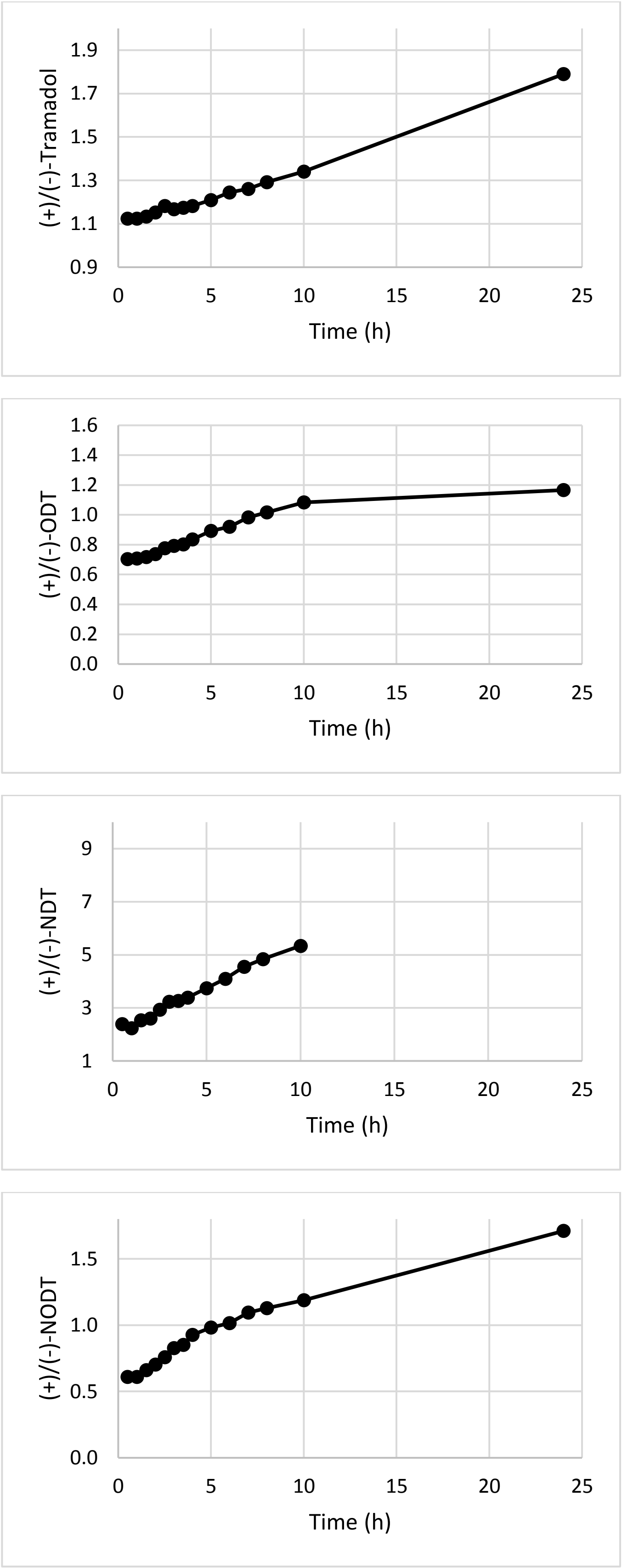

Subject 14 Subject 02

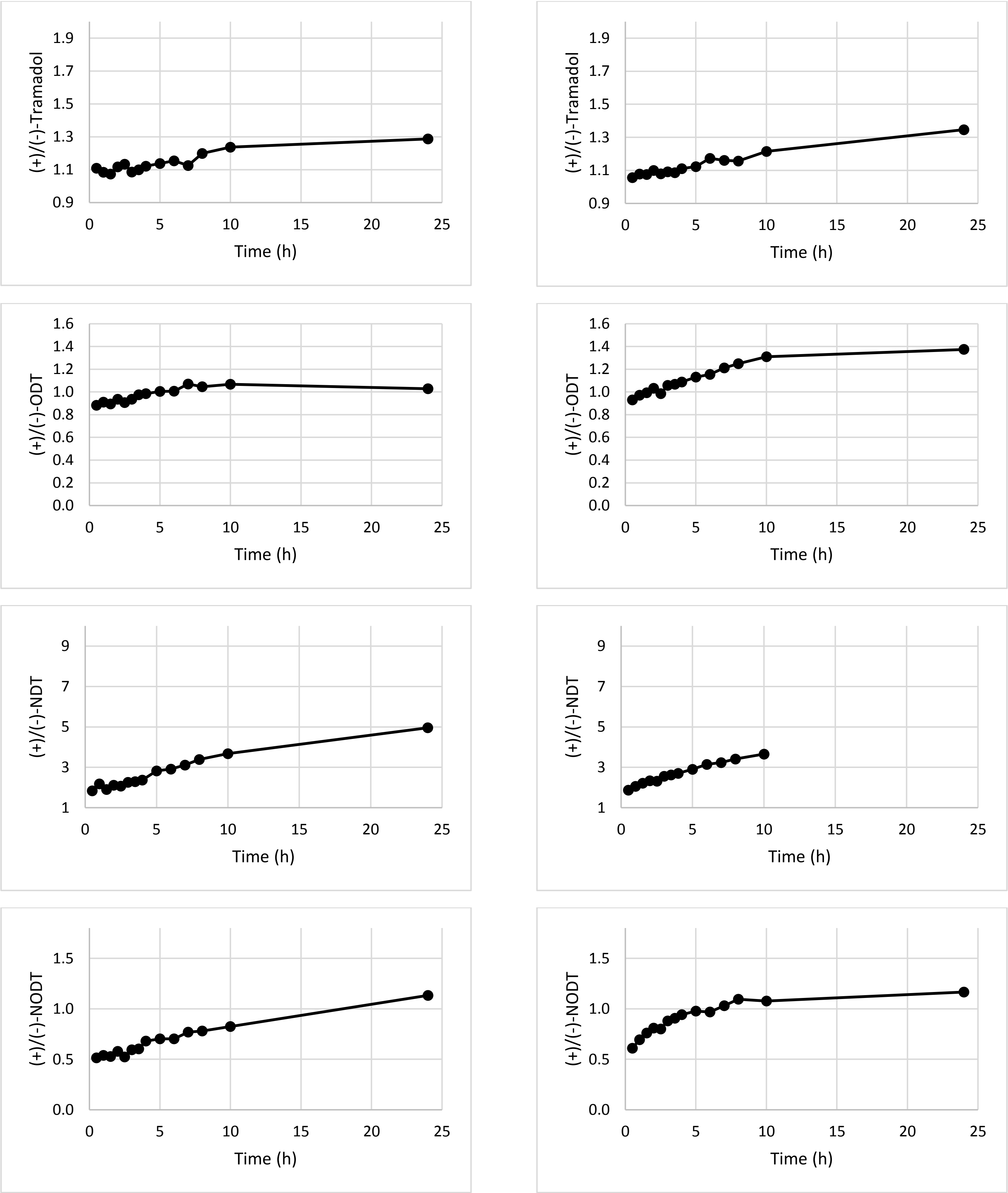

Subject 11

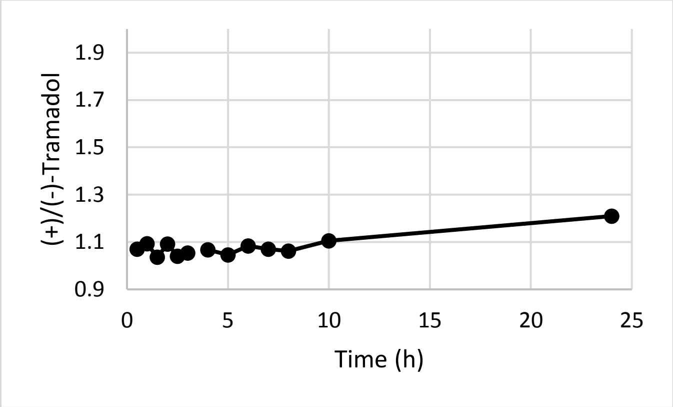

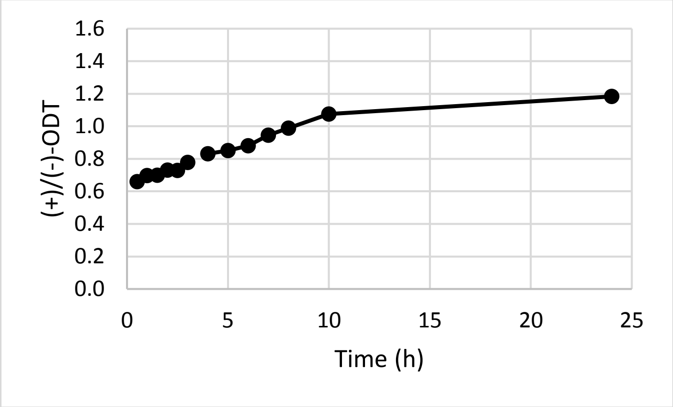

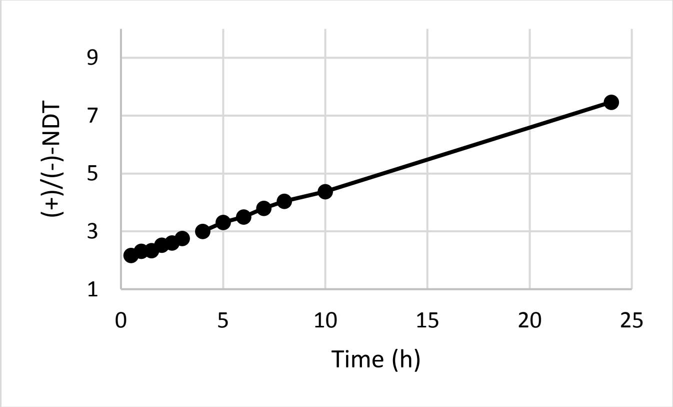

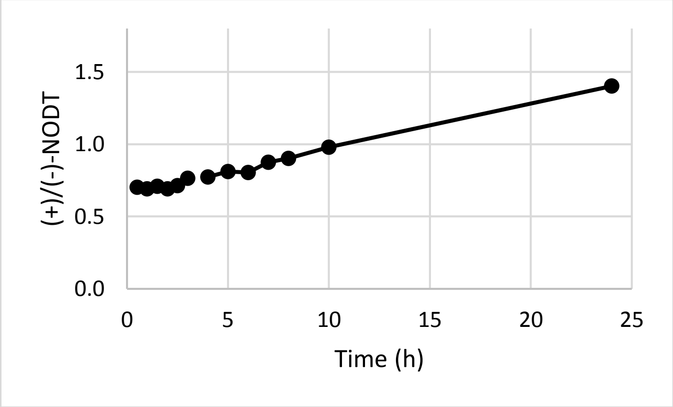

Subject 15

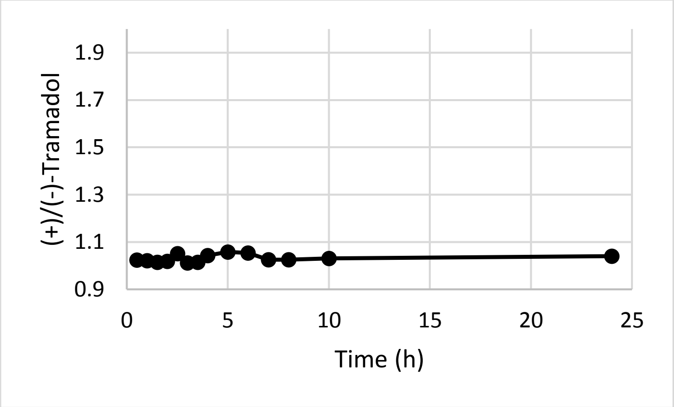

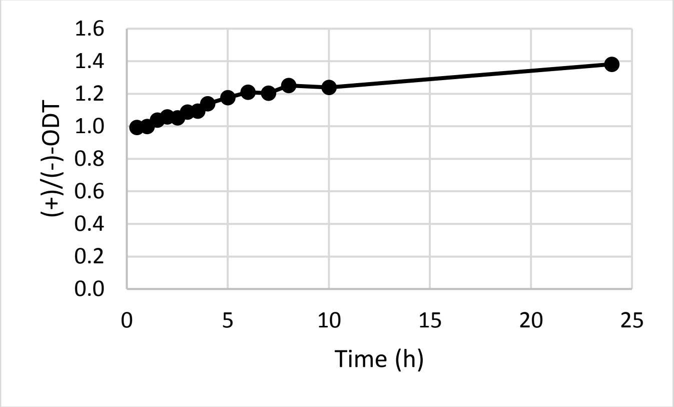

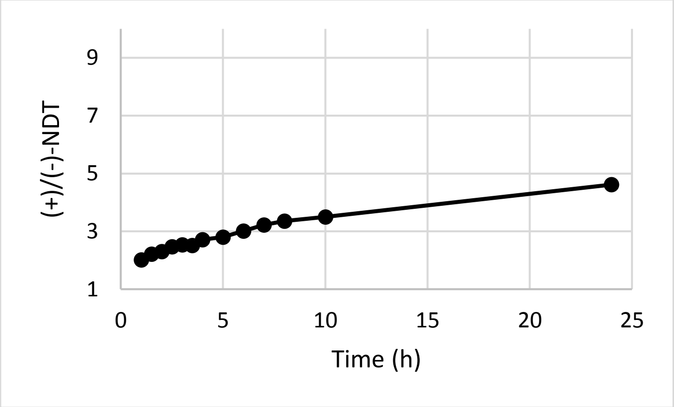

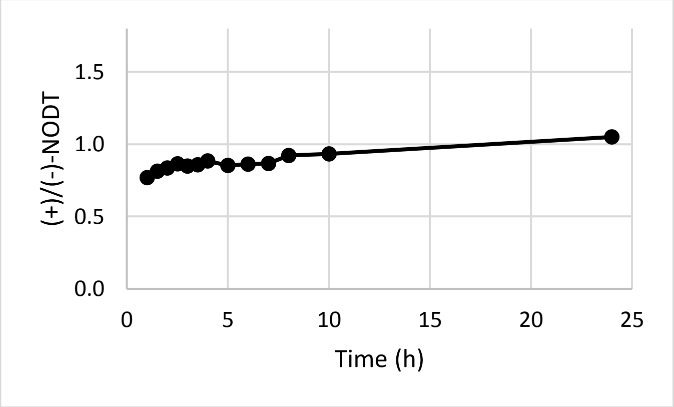

Subject 12

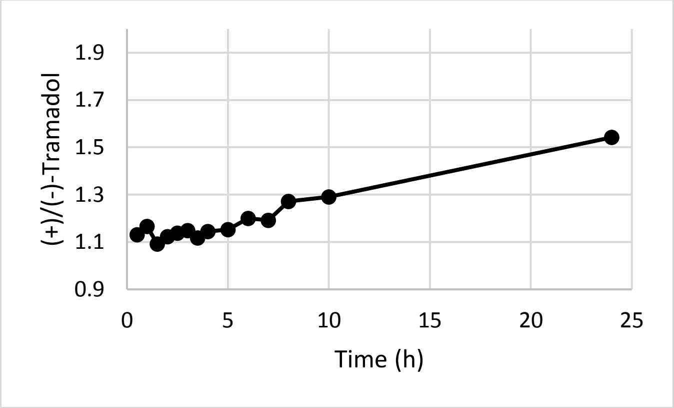

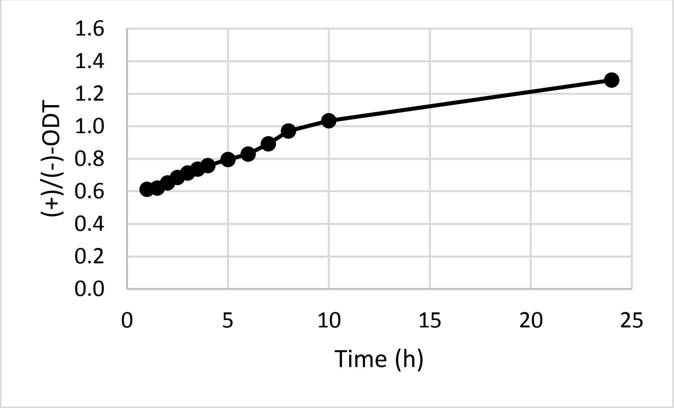

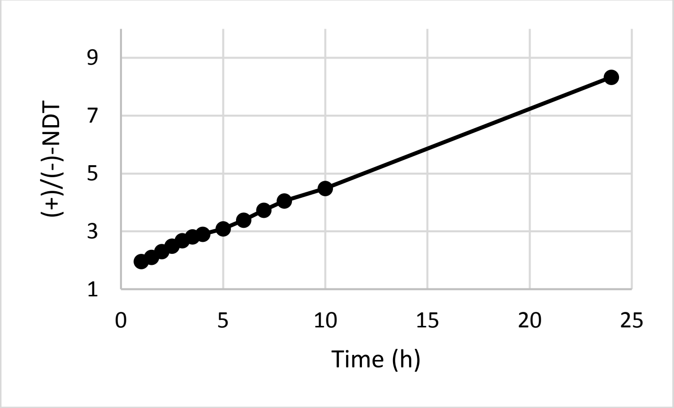

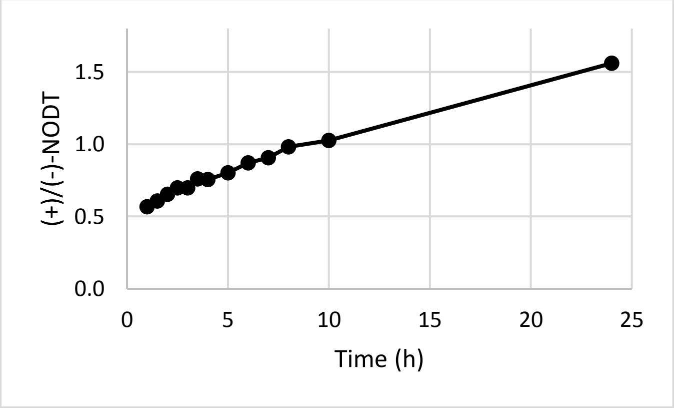

Subject 03

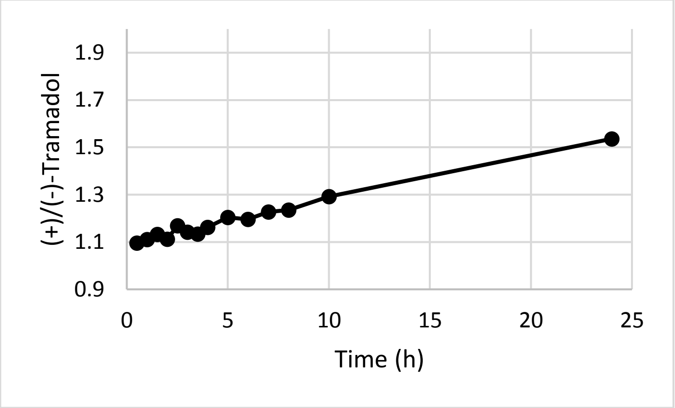

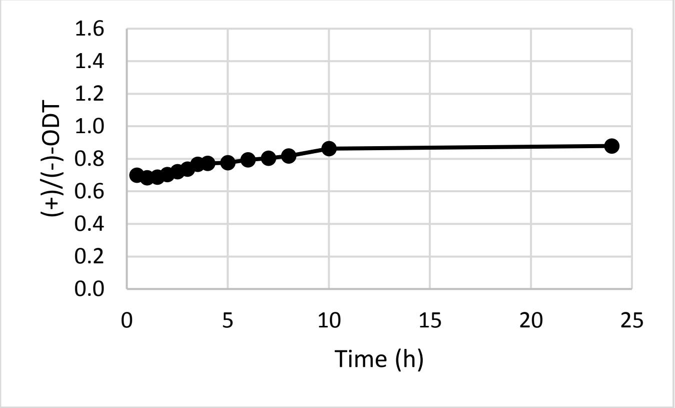

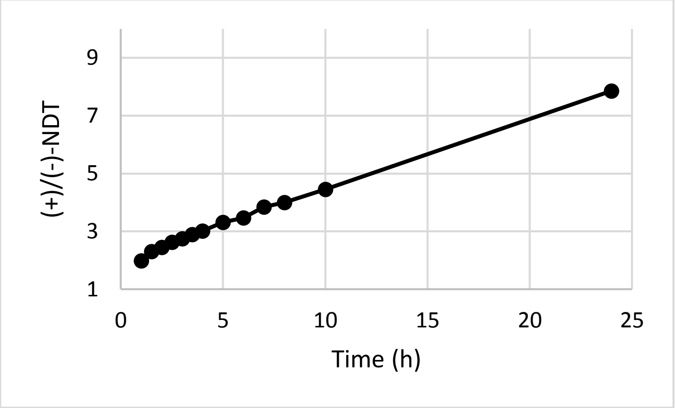

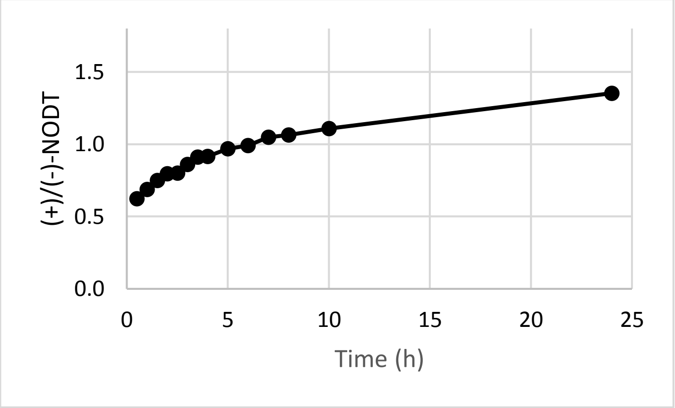

Subject 07

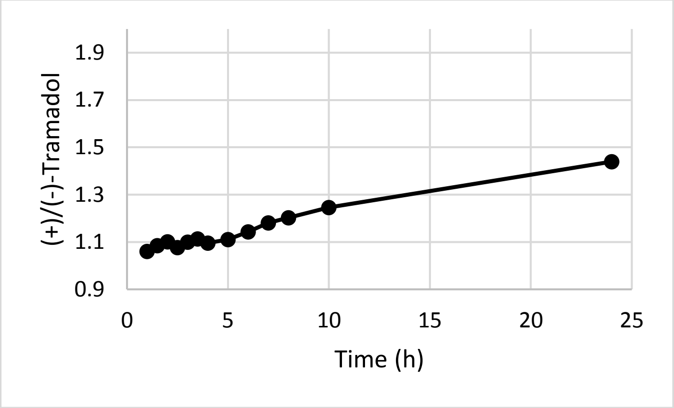

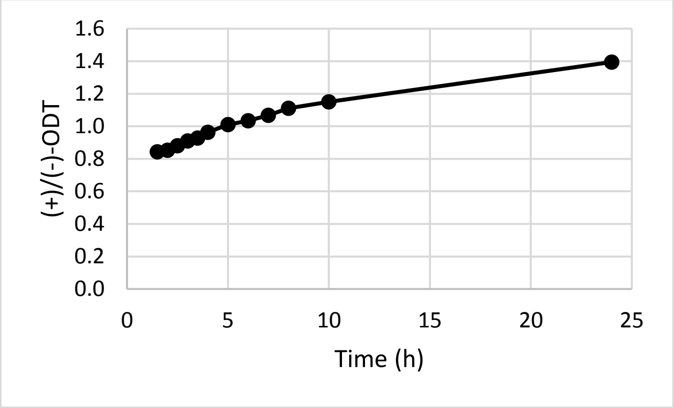

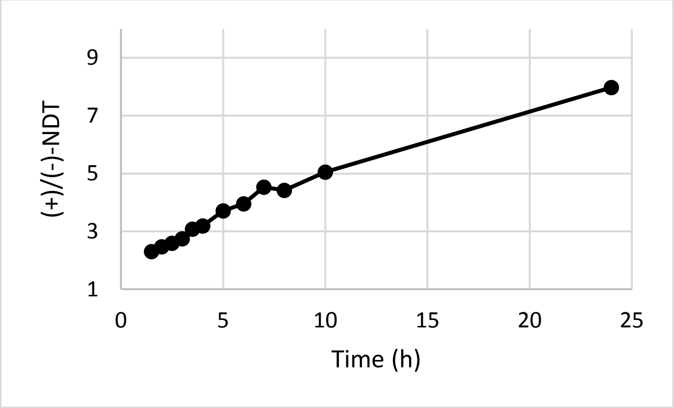

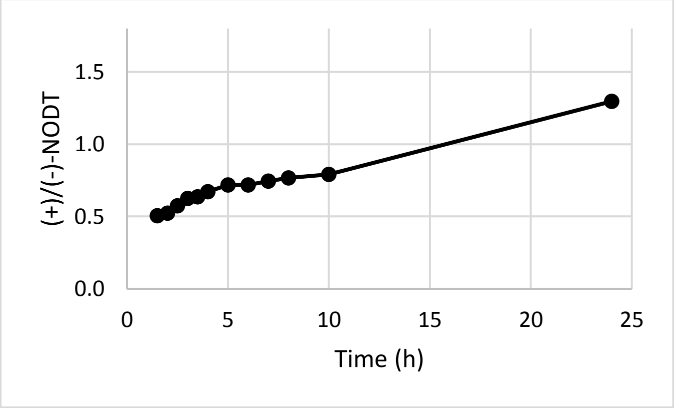

Subject 13

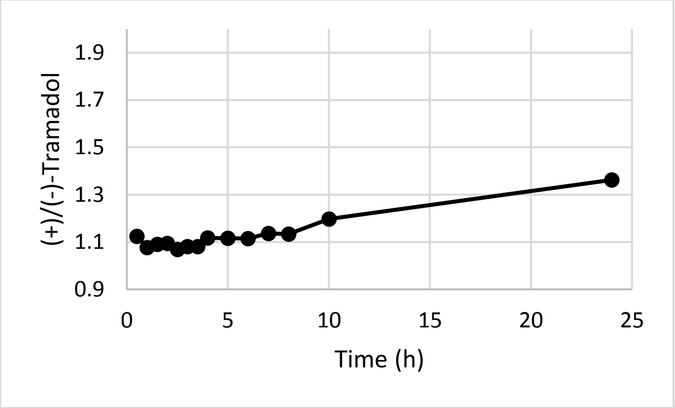

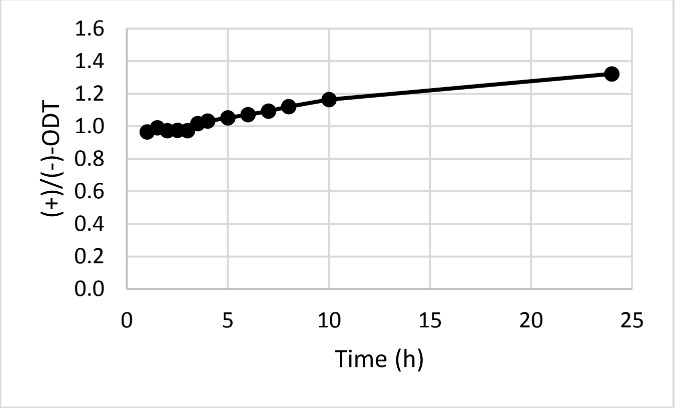

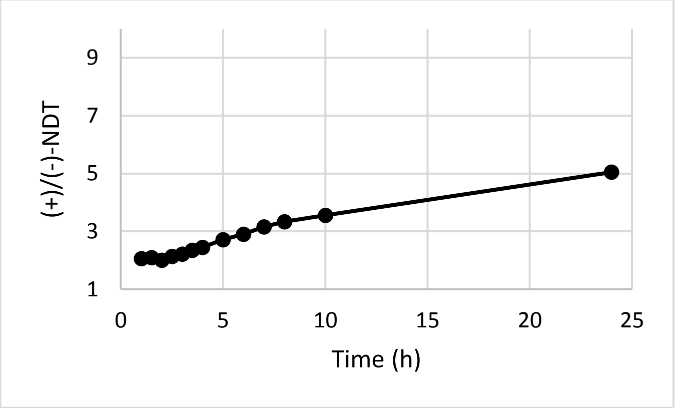

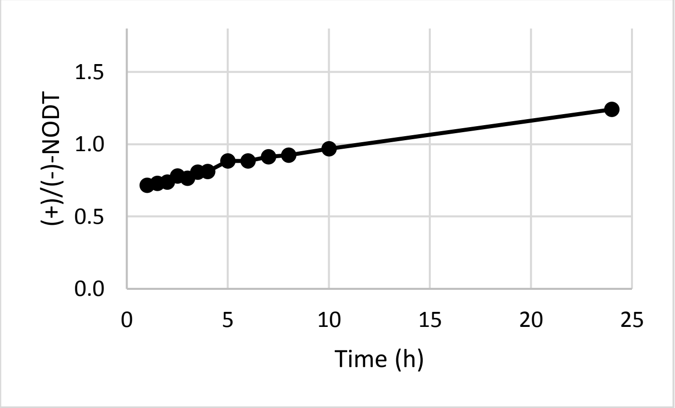

Subject 16 Subject 04

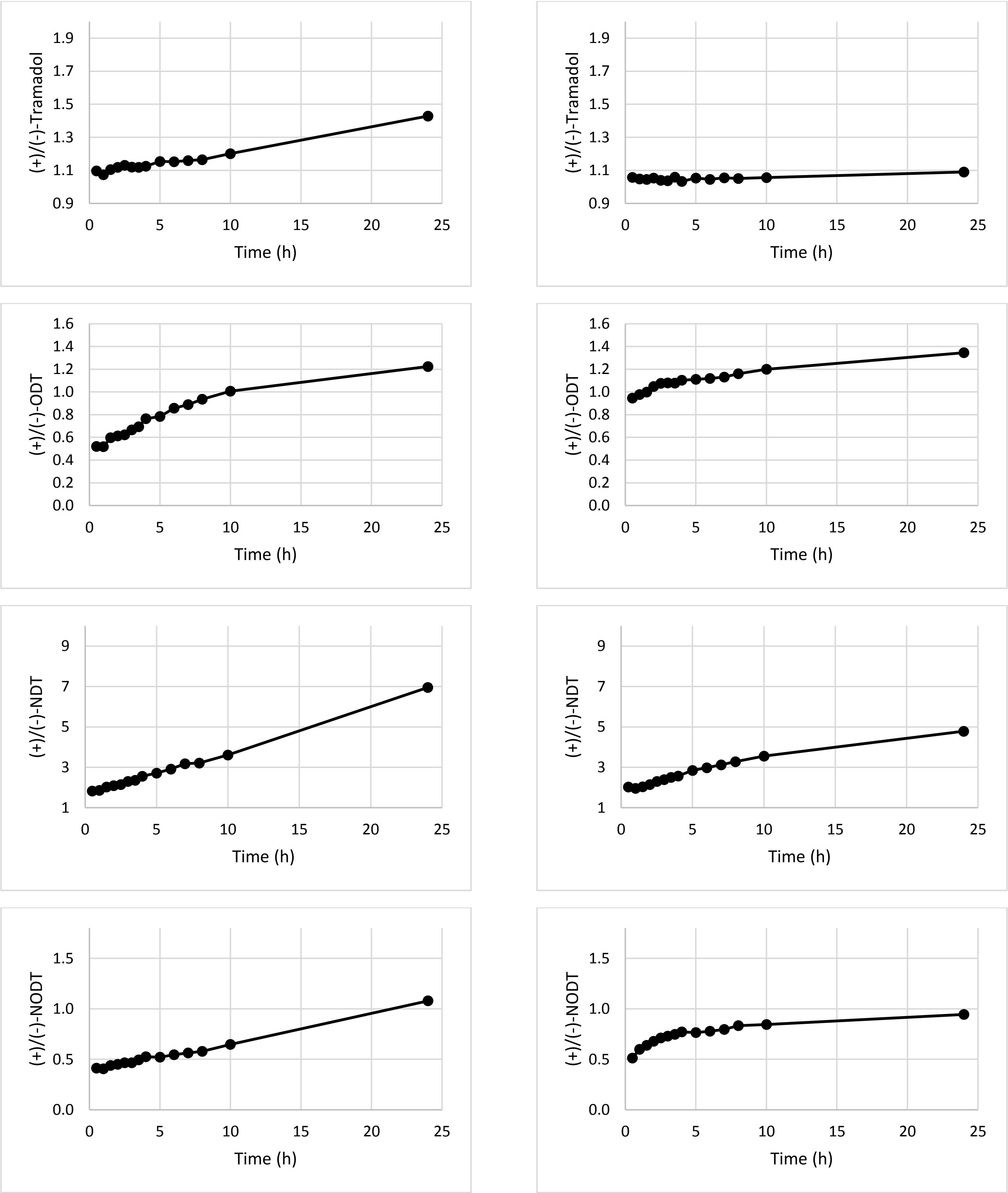

Subject 06

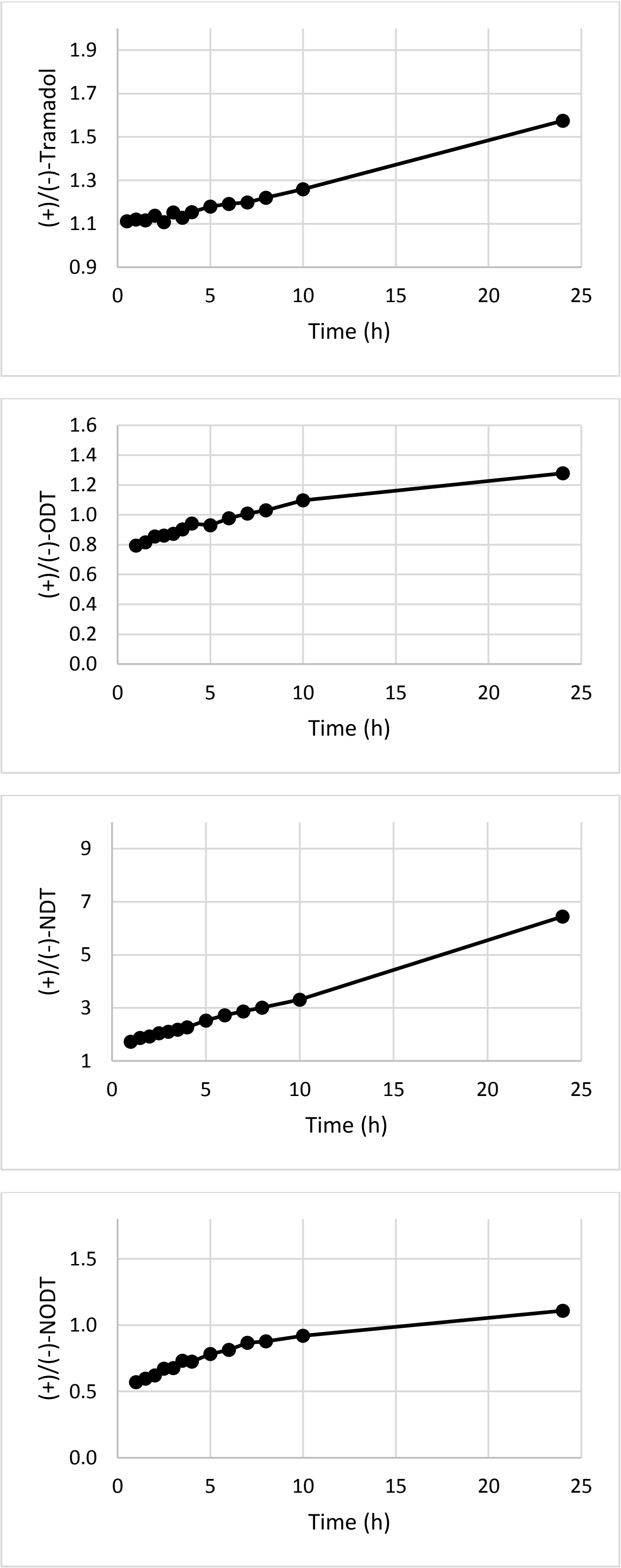

Subject 19

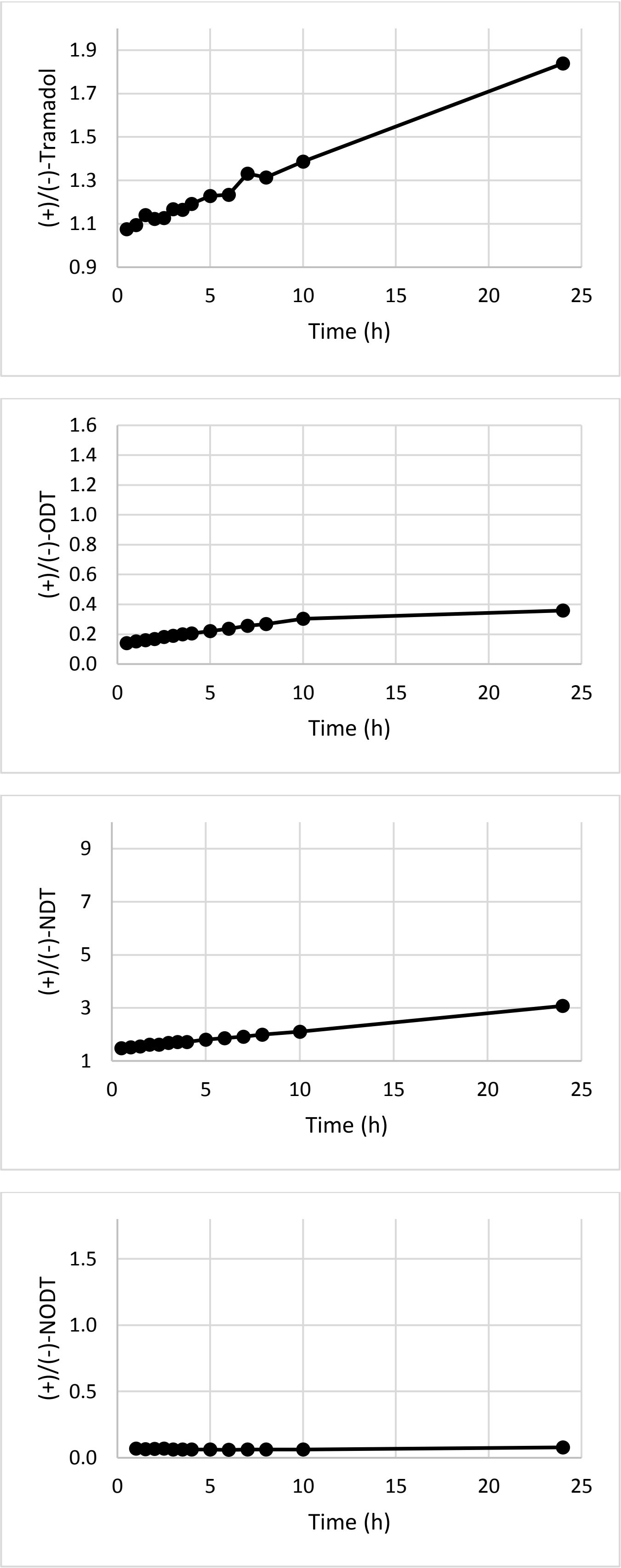

Subject 05

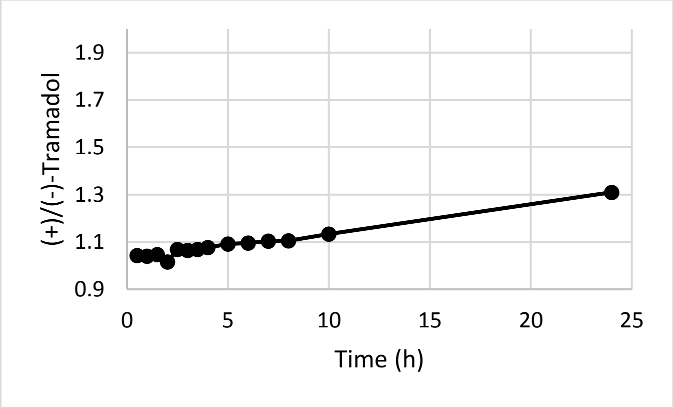
(+)/(-)-Tramadol
1.9
1.7
1.5
1.3
1.1
0.9
0
5
10
15
20
25
Time (h)

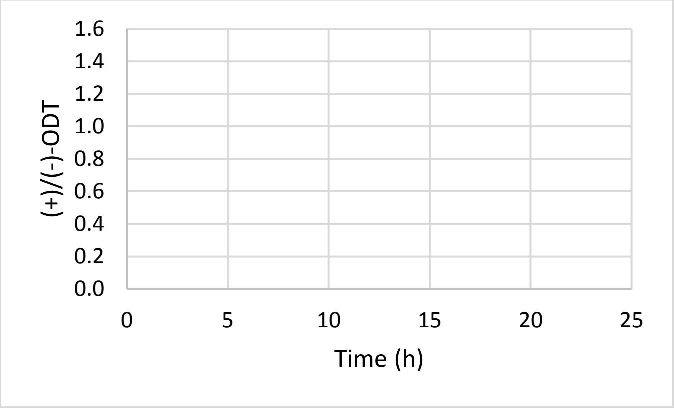
(+)/(-)-ODT
1.6
1.4
1.2
1.0
0.8
0.6
0.4
0.2
0.0
0
5
10
15
20
25
Time (h)

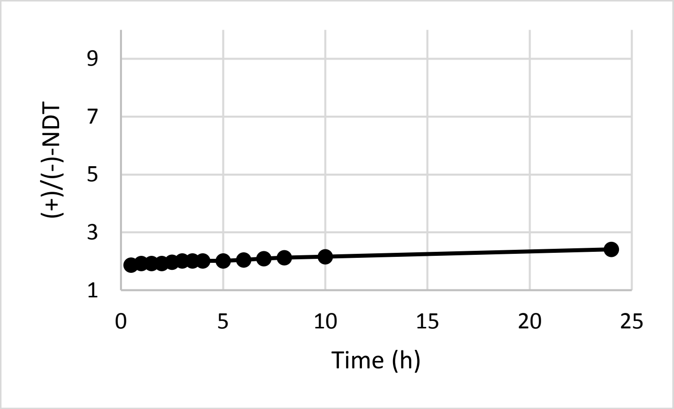
(+)/(-)-NDT
9
7
5
3
1
0
5
10
15
20
25
Time (h)

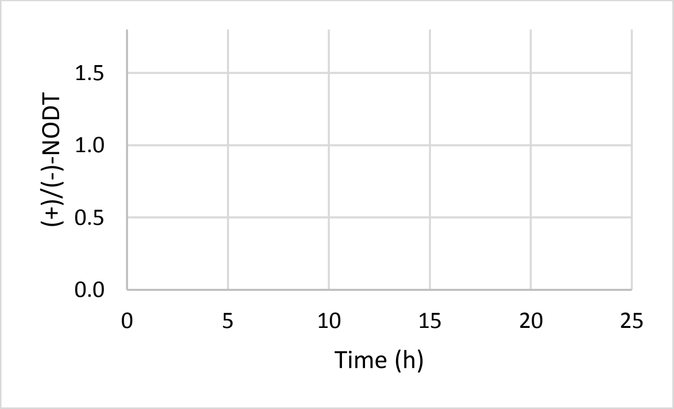
(+)/(-)-NODT
1.5
1.0
0.5
0.0
0
5
10
15
20
25
Time (h)
